# Co-Regulation of Cell Polarization and Migration by Caveolar Proteins PTRF/Cavin-1 and Caveolin-1

**DOI:** 10.1371/journal.pone.0043041

**Published:** 2012-08-13

**Authors:** Michelle M. Hill, Noor Huda Daud, Cho Sanda Aung, Dorothy Loo, Sally Martin, Samantha Murphy, Debra M. Black, Rachael Barry, Fiona Simpson, Libin Liu, Paul F. Pilch, John F. Hancock, Marie-Odile Parat, Robert G. Parton

**Affiliations:** 1 The University of Queensland Diamantina Institute, The University of Queensland, Brisbane, Queensland, Australia; 2 Institute for Molecular Bioscience, The University of Queensland, Brisbane, Queensland, Australia; 3 School of Pharmacy, The University of Queensland, Brisbane, Queensland, Australia; 4 Department of Biochemistry, Boston University School of Medicine, Boston, Massachusetts, United States of America; 5 Centre for Microscopy and Microanalysis, The University of Queensland, Brisbane, Queensland, Australia; Institute of Molecular and Cell Biology, Singapore

## Abstract

Caveolin-1 and caveolae are differentially polarized in migrating cells in various models, and caveolin-1 expression has been shown to quantitatively modulate cell migration. PTRF/cavin-1 is a cytoplasmic protein now established to be also necessary for caveola formation. Here we tested the effect of PTRF expression on cell migration. Using fluorescence imaging, quantitative proteomics, and cell migration assays we show that PTRF/cavin-1 modulates cellular polarization, and the subcellular localization of Rac1 and caveolin-1 in migrating cells as well as PKCα caveola recruitment. PTRF/cavin-1 quantitatively reduced cell migration, and induced mesenchymal epithelial reversion. Similar to caveolin-1, the polarization of PTRF/cavin-1 was dependent on the migration mode. By selectively manipulating PTRF/cavin-1 and caveolin-1 expression (and therefore caveola formation) in multiple cell systems, we unveil caveola-independent functions for both proteins in cell migration.

## Introduction

Caveolin-1 is an integral membrane protein required for formation of small plasma membrane invaginations termed caveolae [1]. Caveolae have been proposed to regulate numerous processes including lipid metabolism, endocytosis and cell migration. The presence of caveolin-1 was assumed to equate to caveolae formation until recent reports that an adapter-like protein, PTRF (polymerase I and transcript release factor), also called cavin-1, is required for formation of caveolae [2], [3]. PTRF/cavin-1 mutations were recently reported in patients with lipodystrophy and muscular dystrophy, correlating with perturbations in caveola function [4], [5] and further supporting a physiological role of PTRF/cavin-1 in caveolae. Related proteins, including SRBC-cavin3 [6] and SDPR-cavin2 [7] have also been reported to regulate caveola endocytosis and membrane tubulation, respectively. In addition, a fourth, muscle-specific member of the family, MURC-cavin4 has been identified [7], [8]. We have examined the ability of each cavin family member to direct caveola formation in the presence of caveolin-1 and showed that when expressed at similar levels, only PTRF/cavin-1 induced the formation of abundant caveolae [8]. These results suggest that PTRF/cavin-1 is likely to be the mediator of caveola formation *in vivo* while the other members regulate other aspects of caveola function such as endocytosis.

A role for caveolin-1 in cell migration has been well-established, mostly through experiments involving manipulation of caveolin-1 expression levels. While some studies report a reduction in directional migration upon loss of caveolin-1, other studies find increased migration (reviewed in [9]). This apparent contradiction may be due to the lack of discrimination between caveolin-1 function within and outside of caveolae. Non-caveolar roles for caveolin-1 are increasingly recognized [10], however, tools for dissecting these functions were not available until the recent discovery of PTRF/cavin-1 as an essential co-factor in caveola formation [2], [3], [11]. We previously reported that expression of PTRF/cavin-1 in prostate cancer PC3 cells reduced transmigration, via a decrease in MMP-9 production independent from de novo caveola formation [12]. This suggests that PTRF/cavin-1 may also have roles independent of caveolae. In the current study, we examined whether PTRF/cavin-1 and caveolin-1 function solely from caveolae during migration. We further utilized the PC3 cell system to explore molecular changes in membrane fractions upon induction of caveola formation.

## Results

### Modulation of PTRF/cavin-1 Expression Affects Cell Migration

**Figure 1:**
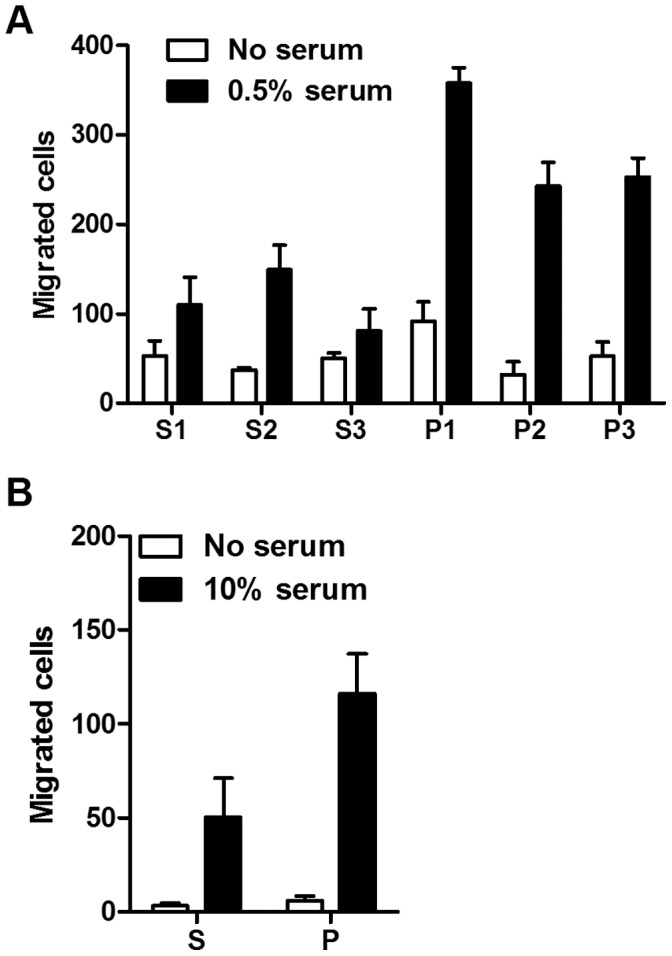
Loss of PTRF/cavin-1 increases transmigration. Transmigration toward the indicated serum concentration in the lower chamber was measured for (A, n = 4, p<0.0001) individual clones of DU145 prostate cancer cells or (B, n = 3, p<0.05) pooled NIH3T3 fibroblasts, stably transfected with scrambled shRNA (s) or PTRF/cavin-1 shRNA (p). Data are shown as mean ± SEM. Two-way ANOVA was used to assess the significance of PTRF/cavin-1 knockdown on migration.

We have previously reported that exogenous expression of PTRF/cavin-1 in the prostate cancer cell line PC3, which expresses abundant caveolin-1 but lacks PTRF/cavin-1, significantly reduced transmigration on collagen-coated polycarbonate filters [12]. To determine the effect of PTRF/cavin-1 expression on independent cell lines, we down-regulated PTRF/cavin-1 in two cell lines using shRNA-mediated knockdown. In agreement with a role for PTRF/cavin-1 in reducing cell migration, chemotaxis to serum was increased in three PTRF/cavin-1 down-regulated prostate cancer DU145 clones, compared to three clones stably transfected with scrambled shRNA (Fig 1A). Furthermore, transmigration of pooled shPTRF/cavin-1 NIH3T3 fibroblasts [2] was increased compared to control knockdown with scrambled shRNA (Fig 1B). Together, the increase in cell migration upon PTRF/cavin-1 knockdown in DU145 and NIH3T3 cells corroborates our previous report of reduced cell transmigration upon PTRF/cavin-1 expression in PC3 cells, which has a natural lack of PTRF/cavin-1 but expresses caveolin-1 [12].

**Figure 2:**
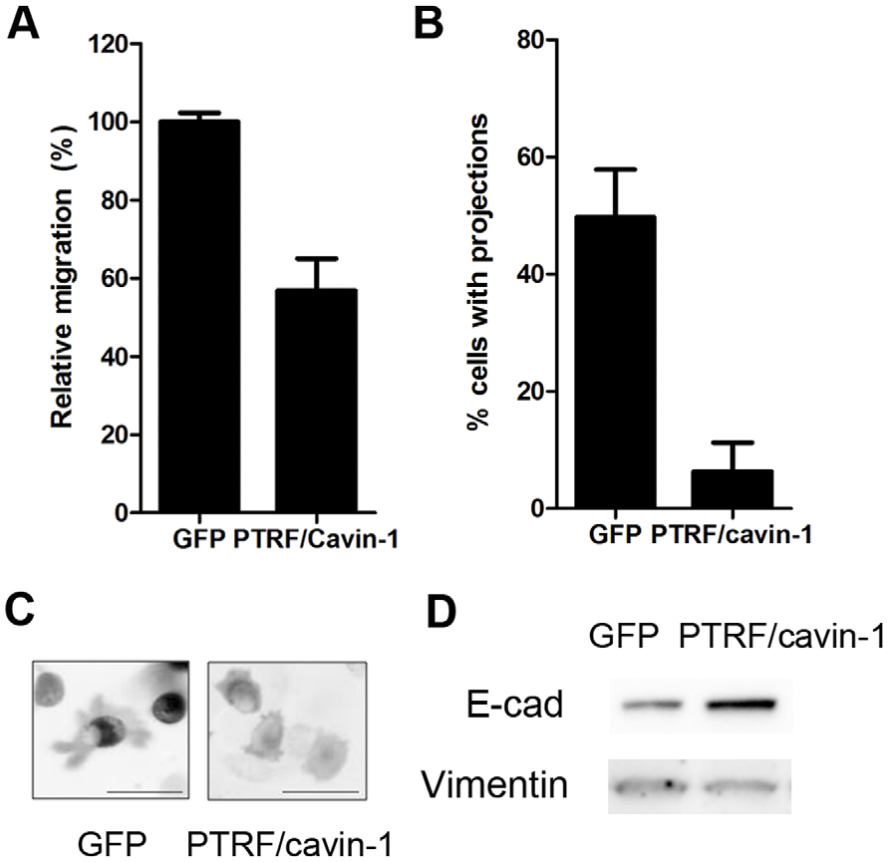
PTRF/cavin-1 expression in PC3 cells reduces 2D migration concomitant with reduced protrusions and mesenchymal epithelial transition. (A) Relative migration in a wound-healing assay was assessed by time-lapse video microscopy as described in materials and methods. P<0.01 (B) Representative still images showing cell morphology. (C) Number of projections was quantitated by three individual researchers in random single cells from three independent videos (N>30 cells assessed per researcher, shown as mean ± SEM, p<0.05). (D) Total cell lysates (20 µg) from GFP or PTRF/cavin-1-GFP PC3 cells were separated by SDS-PAGE and immunoblotted using anti-E-cadherin or anti-vimentin antibodies as indicated. Data representative of 3 independent experiments.

Since differences have been reported between two dimensional (planar) and three-dimensional (through a filter pore) migration systems [13], we verified whether PTRF/cavin-1 expression also reduced two dimensional migration. We examined the migration and morphology of PC3 cells during 2-dimensional migration in a wound-healing assay using time-lapse video microscopy. PC3 cells expressing PTRF/cavin-1-GFP showed a 2-fold reduction in 2-dimensional, random cell migration compared to control cells expressing GFP (Figure 2A). Interestingly, expression of PTRF/cavin-1-GFP in PC3 cells significantly reduced the proportion of cells exhibiting projections (Figure 2B, 2C).

Changes in migratory potential and morphology are associated with epithelial-mesenchymal transition (EMT). Furthermore, we have previously documented that lack of PTRF/cavin-1 expression causes an increase in matrix metalloprotease-9 production [12], which is also a hallmark of EMT. We therefore examined PC3 cells stably transfected with plasmids encoding PTRF/cavin-1-GFP or GFP for expression of two EMT markers, E-cadherin and vimentin. As shown in Figure 2D, PC3 cells stably expressing PTRF/cavin-1-GFP showed an increase in E-cadherin and loss of vimentin expression, in agreement with PTRF/cavin-1 preventing EMT, or driving mesenchymal-epithelial transition (MET). Taken together, these results point towards an inhibitory effect of PTRF/cavin-1 expression on cell migration.

### Polarization of Caveolin-1 and PTRF/cavin-1 during Cell Migration

**Figure 3:**
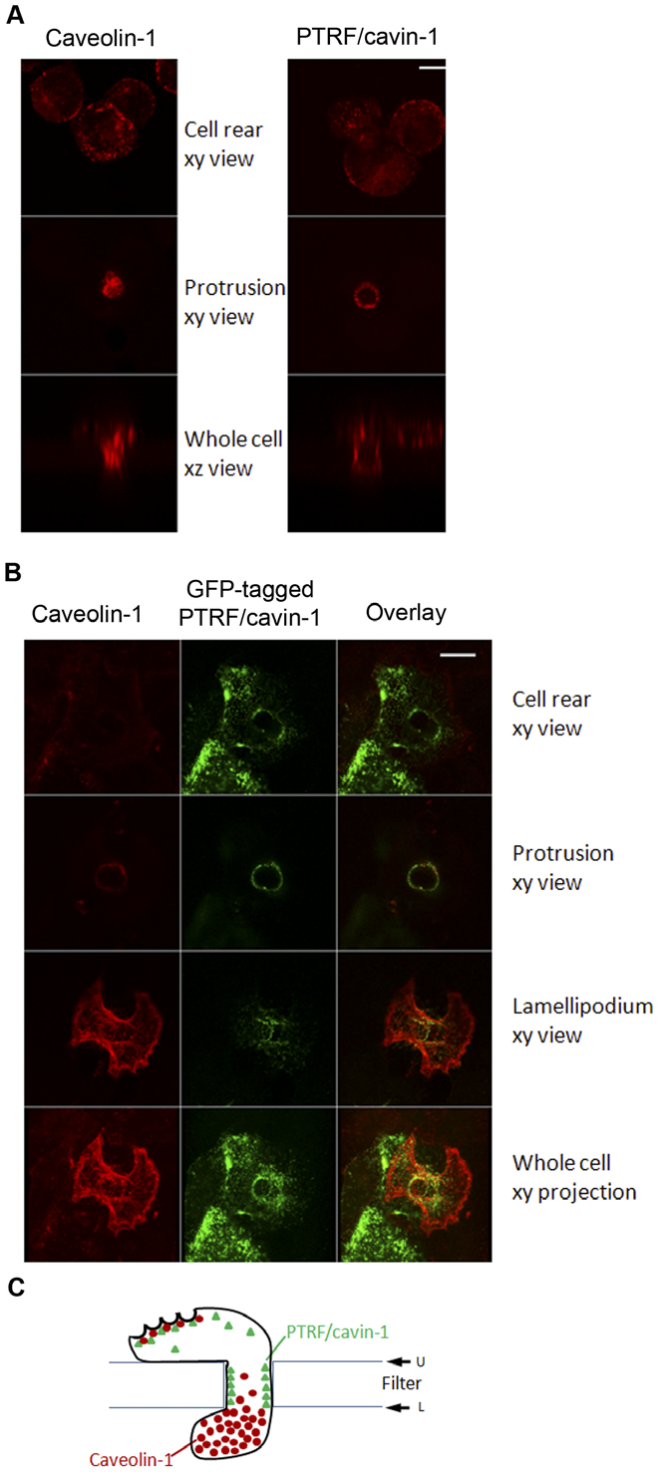
Differential polarization of caveolin-1 and PTRF/cavin-1 during 3D migration. (A) Transmigrating NIH 3T3 cells were fixed and immuno-labeled using anti-caveolin-1 or anti-PTRF/cavin-1 antibodies and imaged by confocal microscopy. Shown are xy planes of cell rear, protrusion through the pore or xz planes through the center of the pore. Bar represents 10 µM. (B) Transmigrating PTRF/cavin-1-GFP expressing PC3 cells were fixed and immuno-labeled using anti-caveolin-1 antibody followed by biotinylated anti-rabbit antibody and texas red avidin. Cells were then imaged by confocal microscopy. Bar represents 10 µM. (C) Schematic representation of the differential caveolin-1 and PTRF/cavin-1 polarization during transmigration.

We have previously reported the polarization of caveolin-1 and caveolae during 3-dimensional (3D) migration in endothelial cells using both confocal and immuno-electron microscopy [13], [14]. Caveolin-1 accumulates in the front protrusion while caveolae accumulate in the rear of the cells [13]. Furthermore, caveolin-1 in the anterior protrusion is associated with vimentin intermediate filaments [15]. To determine the localization of PTRF/cavin-1 during transmigration, NIH3T3 cells traversing the pores of a collagen-coated polycarbonate filter were immuno-labeled with antibodies to caveolin-1 or PTRF/cavin-1. Confocal fluorescence microscopy revealed the presence of both caveolin-1 and PTRF/cavin-1 at the cell rear (Figure 3A, top panel), consistent with a specific localization of caveolae to the rear [13], [14]. Intriguingly, while both caveolin-1 and PTRF/cavin-1 could be detected at the cell protrusion, PTRF/cavin-1 was observed as a distinct ring at the cell-filter contact region (Figure 3A, middle right panel), while caveolin-1 was observed throughout the center of the protrusion as previously noted (Figure 3A, middle left panel).

Next we made use of prostate cancer PC3 cells expressing abundant endogenous caveolin-1, and exogenous PTRF/cavin-1-GFP [2]. Both proteins were detected at the rear of transmigrating cells (Figure 3B), consistent with the presence of caveolae (requiring both proteins) in this subcellular localization. In a fashion similar to NIH3T3 cells, the anterior of the cell showed extensive staining for caveolin-1 through the protrusion, without co-localization of PTRF/cavin-1-GFP. PTRF/cavin-1-GFP was instead observed as a distinct ring at the cell-filter contact site without caveolin-1 (Figure 3B, protrusion xy view). Three-dimensional reconstruction of the confocal images further illustrates the distinct localization of caveolin-1 and PTRF/cavin-1 in the protrusion and lamellipodium extending underneath the lower filter side (Video S1). Hence our data support caveolar-dependent and caveolar-independent localization of caveolin-1 and PTRF/cavin-1 at discrete locations during transmigration in two different cell models: NIH3T3 fibroblasts and metastastic prostate cancer cells (PC3). To our knowledge, this is the first report of a caveolar-independent, membrane localization for PTRF/cavin-1.

**Figure 4:**
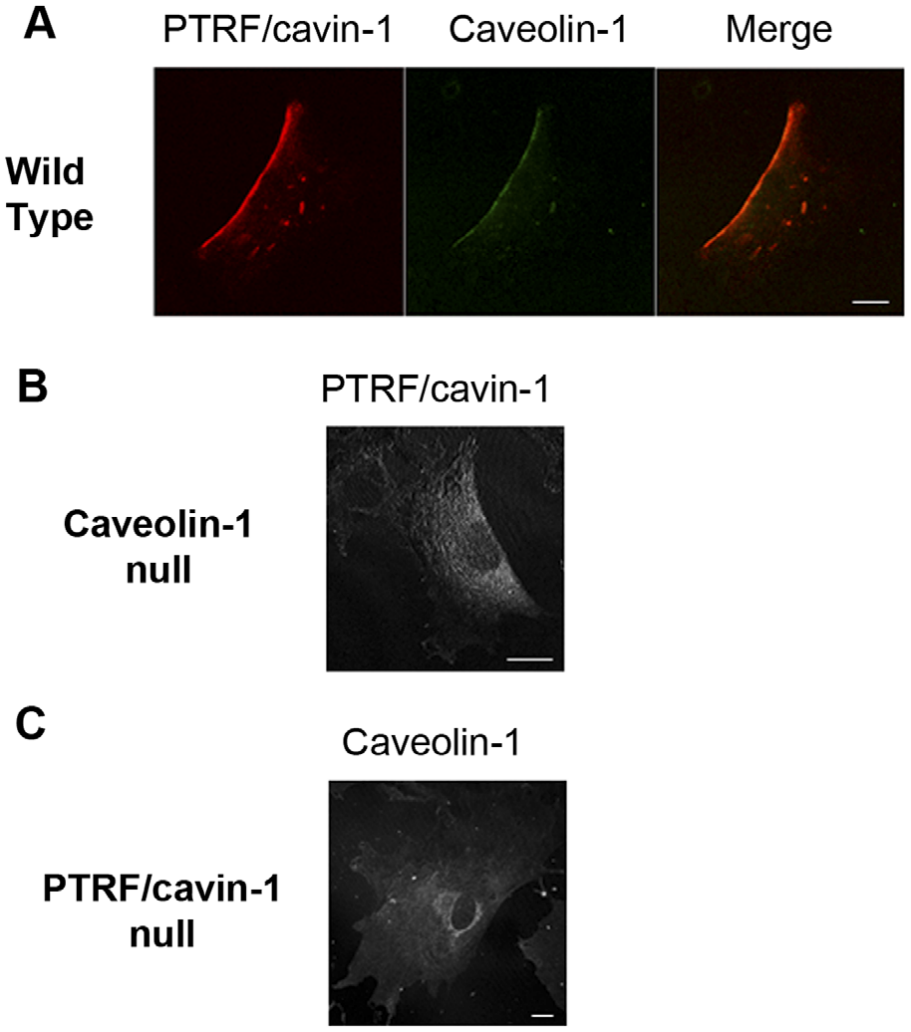
Inter-dependent Polarization of caveolin-1 and PTRF/cavin-1. Wild type (WT), caveolin-1^−/−^ and PTRF/cavin-1^−/−^ mouse embryo fibroblasts (MEF) were plated on coverslips at low density. Immunostaining was performed with (A) mouse anti-caveolin-1 and rabbit anti-PTRF, (B) rabbit anti-PTRF/cavin-1, or (C) rabbit-anti-caveolin antibody respectively. Randomly migrating cells are imaged. Bar, 20 µM.

Polarization of both proteins was further assessed in a two dimensional, scratch wound assay. Wild type mouse embryo fibroblasts exhibited posterior polarization of caveolin-1 typical in this model (Figure 4A). PTRF/cavin-1 was co-localized with caveolin-1 in the cell rear, consistent with caveolae posterior accumulation in two-dimensional migrating cells [13]. When PTRF/cavin-1 immuno-staining was performed on 2D migrating mouse embryo fibroblasts isolated from caveolin-1 gene disrupted mouse, polarization of PTRF/cavin-1 was lost (Figure 3B). Reciprocally, caveolin-1 accumulation at the rear of 2D migrating cells was lost in MEFs from PTRF/cavin-1 knock out mouse (Figure 3C). These results indicate that in flat migrating cells, caveolin-1 and PTRF/cavin-1 regulate each other’s accumulation at the rear of the cell body, presumably in the form of caveolae.

### Caveolae-dependent and –Independent Functions of Caveolin-1 in Migration

**Figure 5:**
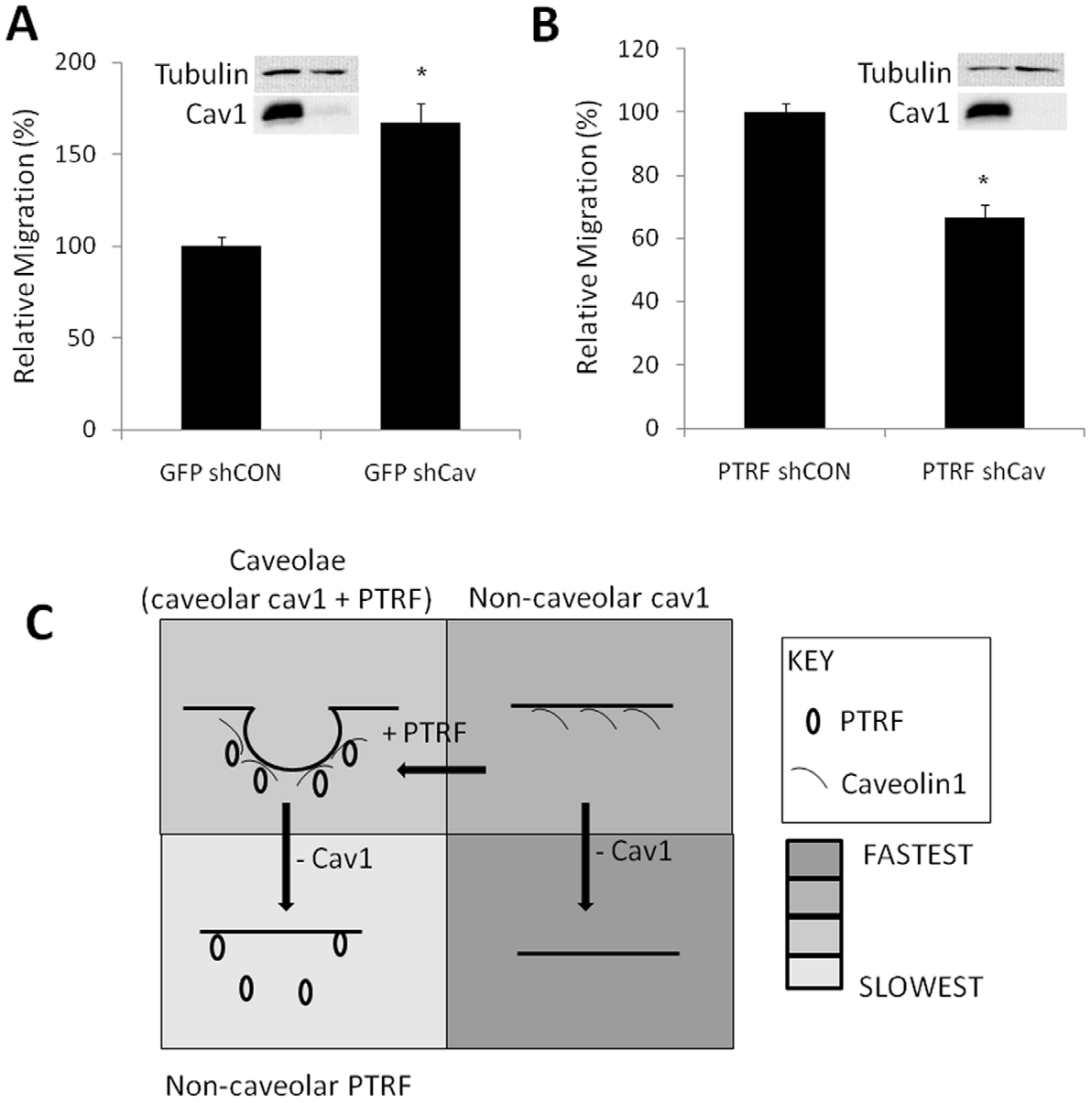
Distinct roles for caveolar and non-caveolar caveolin-1 in cell migration. (A) Knockdown of caveolin-1 in GFP PC3 cells increases transmigration. (B) Knockdown of caveolin-1 in PTRF/cavin-1-GFP cells reduces rate of transmigration. N = 3, shown as mean ± SEM. p<0.00001. (C) Diagrammatic summary of the results.

To distinguish between caveolae-dependent and -independent roles of caveolin-1 and PTRF/cavin-1, we used lentivirus-mediated shRNA to down-regulate caveolin-1 in PC3 lines stably expressing GFP or PTRF/cavin-1-GFP. Control lentiviral constructs with a scrambled sequence or against the human papilloma virus E7 protein, which is not expressed in PC3 cells, were used independently and produced similar results. The loss of non-caveolar caveolin-1 in GFP-expressing PC3 cells (PTRF/cavin-1-negative) led to an increase in transmigration (Figure 5A), suggesting that non-caveolar caveolin-1 performs an anti-migratory function. In contrast, down-regulation of caveolar caveolin-1 in PTRF/cavin-1-GFP-expressing PC3 cells caused a reduction in transmigration (Figure 5B). These results suggest that caveolin-1 in caveolar or non-caveolar compartments has distinct functions.

### Rac1 Subcellular Localization is Modulated by PTRF-GFP Expression in PC3 Cells

**Figure 6:**
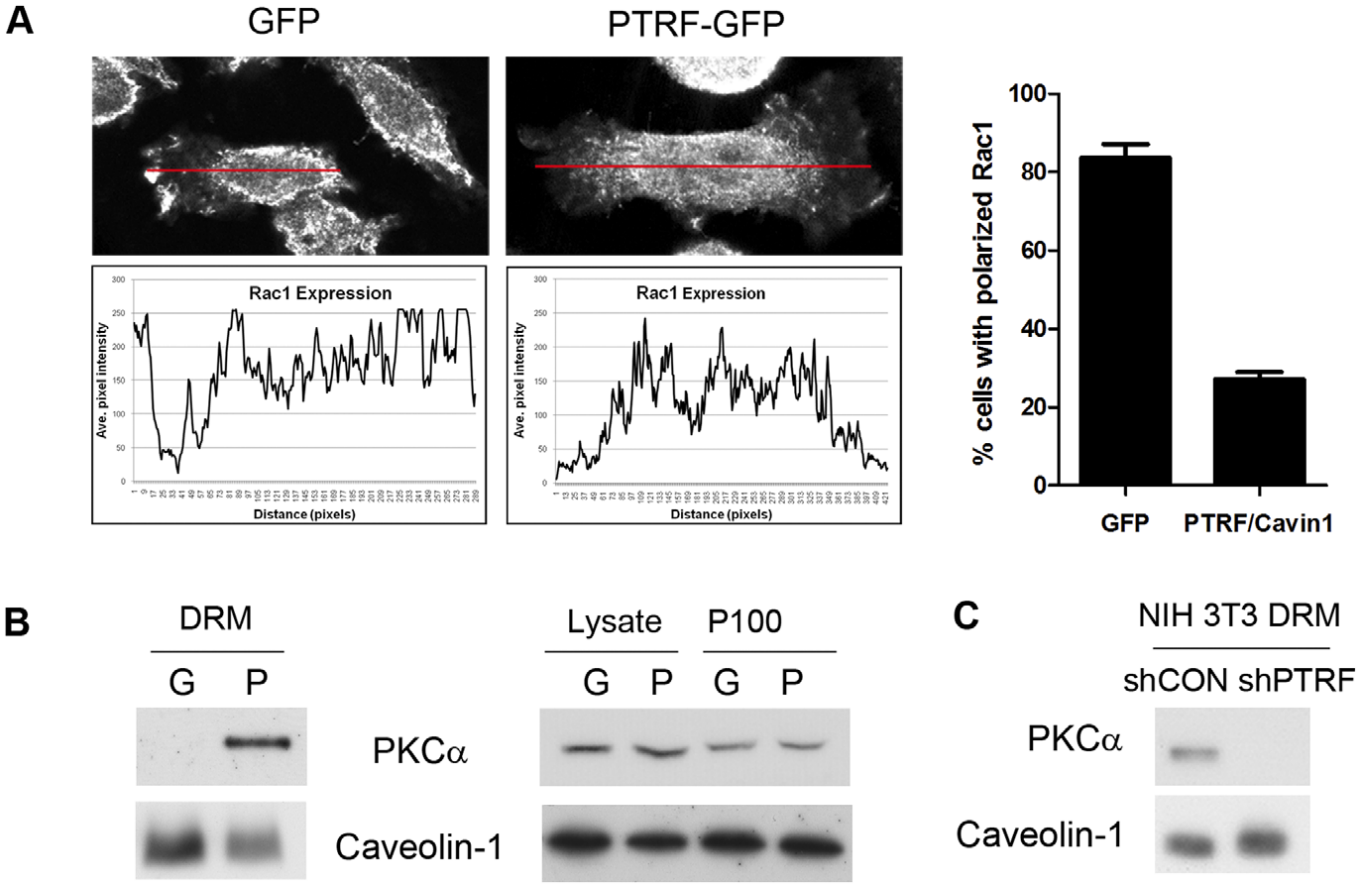
PTRF/cavin-1 expression in PC3 cells impairs Rac1 polarization, but leads to recruitment of PKCα to detergent-resistant membrane (DRM) fraction. (A) Rac1 polarization was examined by confocal immunofluorescence microscopy. Polarization was measured by fluorescence intensity along the length of the cell as indicated by red lines shown. For quantitation, 20 cells over 3 independent experiments were analysed, and Rac1 was deemed polarized when staining was confined to terminal one third of the cell body. P<0.0001. (B) PTRF/cavin-1-GFP expression in PC-3 cells caused recruitment of PKCα to the lipid raft (DRM) fraction, without altering total cellular level or total membrane (P100) level. (C) Loss of PKCα from lipid raft (DRM) fraction in NIH3T3 fibroblasts with PTRF/cavin-1 knockdown.

Loss of caveolin-1 is known to affect cell polarization and migration via Rho family small GTPases, including Rho, Rac and cdc25 [16]. We compared the subcellular localization of Rac1 in PC3 cells stably expressing GFP or PTRF/cavin-1-GFP using immunofluorescence microscopy. We observed a striking loss of Rac1 anterior polarization upon PTRF/cavin-1-GFP expression in PC3 cells, leading to a diffuse staining (Figure 6A). Image analysis and quantitation revealed a significant reduction in Rac1 polarization upon PTRF/cavin-1 expression in PC3 cells (Figure 6B).

We hypothesized that loss of polarization was in part due to caveola formation upon PTRF/cavin-1 exogenous expression. Previous studies revealed that PTRF/cavin-1-GFP expression altered the partitioning of cytoskeletal proteins to lipid raft fractions [17]. Similarly, we now report that expression of PTRF/cavin-1-GFP induces proteomic changes in the total membrane P100 fraction. Statistical analysis using a p-value of 0.05 as cut off revealed that 140 proteins are significantly altered by PTRF/cavin-1-GFP expression, with an additional 34 proteins falling between p-values of 0.05 and 0.1. Rac1 is in the latter category (p = 0.07965) with a PTRF/cavin-1-GFP:GFP SILAC ratio of 0.42±0.09 indicating that PTRF expression in PC3 cells causes a 2.3 fold decrease of Rac1 in the total membrane fraction, confirming our fluorescence microscopy observations.

### Expression of PTRF/cavin-1 Induces the Recruitment of PKCα to Caveolae

By screening the P100 fraction for proteins significantly altered by PTRF/cavin-1 expression, we further identified ten proteins previously implicated in polarization or cell migration, including three adapter proteins of the 14-3-3 family, cytoskeletal linkers (cofilin-1, filamin A), and adhesion proteins (integrins α2, α3, β1, JAM1 and vinculin). This list of proteins, together with Rac1 and caveolin-1, was used to generate a protein interaction network using 2-step shortest paths in GeneGo, which revealed two new hubs, namely protein kinase C (PKC) and Src.

Both PKC and Src have been localized in caveolae/DRM fractions [18], [19], and regulate caveolae structure and function [20], [21]. Reciprocally, their activity is regulated by microdomain localization [22], [23]. However, while Src localization to caveolae requires its acylation [19], [24], PKCα targeting to caveolae appears to be mediated through interaction with SDPR/cavin-2 [18]. Furthermore, cellular polarization [25] and MMP-9-mediated cell migration [26] are both controlled by PKCα. We have previously reported the loss of PKCα from detergent-resistant membrane (DRM) fraction of caveolin-1 null MEFs [2], hence we chose to further investigate PKCα recruitment to caveolae upon expression of PTRF/cavin-1 in PC3 cells.

In contrast to MEFs, PKCα is absent from DRM of control GFP-PC3 cells (Figure 6B). Expression of PTRF/cavin-1-GFP in PC3 cells caused a marked recruitment of PKCα to the DRM fraction (Figure 6B), concomitant with the reported induction of caveola formation [2]. Overall PKCα expression in the cell lysate or P100 fractions was unaltered (Figure 6B). These results suggest that PKCα is specifically recruited to caveolae when PTRF/cavin-1 and caveolin-1 are both expressed. To confirm this result, we utilized NIH3T3 fibroblasts with stable knockdown of PTRF/cavin-1 (shPTRF), which lack caveolae as we previously observed by electron microscopy [2]. In agreement with its caveola-specific recruitment, PKCα was detected in the DRM fraction of control NIH3T3 cells (shCON) but not shPTRF NIH3T3 cells (Figure 6C).

## Discussion

Combining multiple cell lines and migration assays, this study is the first to show inter-dependent polarization of caveolin-1 and PTRF/cavin-1 in migrating cells, and to demonstrate that PTRF/cavin-1 expression regulates cell migration. It further unveils that caveolin-1 plays a different role in cell migration depending on the presence or absence of PTRF/cavin-1 (and thus of caveolae).

Caveolin-1 was previously known to regulate cell migration and polarization. We now show that caveolin-1 polarization is accompanied by PTRF/cavin-1 polarization. In two dimensional migrating cells, both proteins accumulate in the cell rear, where caveolae are also known to accumulate [13]. Furthermore, caveolae seem to be required for the polarization of both PTRF/cavin-1 and caveolin-1, since conditions where the cells lack caveolae result in polarization loss. In 3D migrating cells, we have been able to visualize non caveolar caveolin-1 (throughout cytoplasmic protrusions of trans-migrating cells) but also non caveolar PTRF/cavin-1 (as a ring where the protrusion contacts the filter pore). The mechanism by which PTRF/cavin-1 can localize to non-caveolar membrane is currently unknown. PTRF/cavin-1 binds phosphatidylserine *in vitro* [2], thus one possibility for caveola-independent PTRF/cavin-1 membrane recruitment is an enrichment, or distinct organization, of phosphatidylserine at the plasma membrane-filter (and possibly extracellular matrix) points. Polarization of PTRF/cavin-1, caveolin and caveolae in transmigrating cells is summarized in Figure 3C.

Summarizing what we currently know about the effect of PTRF/cavin-1 expression on cell migration, a mechanism emerges through which by allowing caveola formation and recruitment of caveolar proteins to plasma membrane microdomains, PTRF/cavin-1 allows caveola recruitment of PKCα, modulates cell polarization, decreases MMP-9 production and ultimately cell migration. Multiple pathways are likely to be involved in mediating the effect of PTRF/cavin-1 on cell migration. It is interesting to note that like the phenotype of caveolin-1-null mice, the phenotype of PTRF/cavin-1-null mice does not seem to encompass major defects in cell migration, and regulation must therefore be either compensated for, subtle, or apparent only upon challenge. PKCα targeting to caveolae was previously reported to be mediated through interaction with SDPR/cavin-2 [18]. However, PC3 cells do not express SDPR/cavin-2, or PRKCDBP/cavin-3 [8], both of which are known to bind to PKC isoforms. Hence PTRF/cavin-1 induced DRM-recruitment of PKCα likely occurs via a novel mechanism.

Studies on caveolin-1 and caveolae function commonly employ caveolin-1 over-expression or knockdown approaches. The present data suggest that interpretation of such experiments should to be revisited with knowledge of the PTRF/cavin-1 expression status. Indeed, expression and subcellular localization of these caveolar adapter proteins, are co-regulated with caveolin-1. Furthermore it seems that both caveolin-1 and PTRF/cavin-1 can function from non-caveola locations, as reported here. As illustrated in Figure 5C, the loss of caveolin-1, (presumably via loss of caveolae) releases PTRF/cavin-1 which can function at extra-caveolar locations. Conversely, ectopic expression of caveolin-1 in PTRF (and caveola)-null cells may lead to extra-caveolar caveolin-1 functions. Over-expression of caveolin-1 in PTRF/cavin-1-positive cells would not only increase caveola density, but could also deplete extra-caveolar cavins, and/or increase extra-caveolar caveolin-1 function. Notably, cavins show tissue-specific expression patterns that partially parallel caveolins [2], [8]. Furthermore, caveolin-1 expression in some caveolin-1-negative cell lines does not induce PTRF/cavin-1 expression [27], suggesting that both caveolin-1 and PTRF/cavin-1 expression are limiting for caveola formation in a cell- and tissue- specific manner. Clearly, these previously unrecognized parameters confound the interpretation of experiments. Thus distinct extra-caveolar functions of caveolin-1 and PTRF/cavin-1 reveal a new paradigm in caveolin biology and calls for re-interpretation of the numerous studies on caveolin-1 and caveola function.

The manipulation of PTRF/cavin-1 and caveolin-1 levels in different cell lines allowed us to identify distinct effects of these proteins on cell migration and polarization. Heterologous expression of PTRF in caveolin-1-positive, PTRF/cavin-1-negative PC3 cells results in caveola formation, and reduced cell migration. Down-regulating caveolin-1 in (PTRF/cavin-1-negative) PC3 cells to reduce non-caveolar caveolin led to an increase in cell migration. In contrast, knockdown of caveolin-1 in PTRF/cavin-1-expressing PC3 cells reduced cell migration. These results, summarized in Figure 5C, indicate that caveolin-1 can regulate cell migration without being part of caveolae and functionally build on previous data obtained via imaging [13]. Future studies will establish whether other functions previously ascribed to caveolae [28], [29], [30], [31], [32], [33], [34], [35], [36], [37], [38], [39], [40], [41] may be due to non-caveolar caveolin. Overexpression of caveolin-1 in a PTRF/cavin-1 null breast cancer cell line, SK-BR-3 was recently reported to elicit formation of long tubules [27] that may mediate signaling events different from caveolae.

Distinct non-caveolar functions of caveolin-1 will also have implications for our understanding of cancer development and progression since caveolin-1 mutation or overexpression is suggested to be involved in several cancers [34], [35], [42], [43], [44], [45], [46], [47], [48], [49], [50], [51]. It is now clear that not only the levels of caveolin-1, but also the relative levels of PTRF/cavin-1, must be taken into account when interpreting the role of caveolin-1 in specific disease contexts.

## Materials and Methods

### Antibodies

Caveolin-1 and Rac-1 antibodies were from BD Biosciences. Antibodies to E-cadherin and vimentin were from Cell Signaling Technology. Rabbit anti-PTRF antibodies were produced against a peptide comprising the C-terminal 14 amino acids of mouse PTRF and affinity-purified.

### Cell Culture

Cell culture and western blotting were performed as previously described [2], [8]. NIH3T3 cells with reduced PTRF/cavin-1 expression have been previously generated [2]. DU145 cell lines with reduced PTRF/cavin-1 expression were generated using the same shRNA plasmids, except individual clones rather than pooled population were characterized. PTRF/cavin-1 null murine embryonic fibroblasts have been previously described [3]. PC3 cells lines with down-regulated caveolin-1 were generated using lentiviral stocks containing shRNA to caveolin-1 obtained from Sigma (Mission®). Briefly, PC3 cells expressing GFP or PTRF/cavin-1-GFP were seeded in 96 well plates and infected with lentiviral stocks as per manufacturer’s recommendations. Lentivirus with shRNA against a scrambled sequence (Sigma Mission®) or against the human papilloma virus E7 protein (not expressed in PC3 cells, kind gift from Prof Nigel McMillan, The UQ Diamantina Institute), were used as controls. After infection, cells were selected in puromycin for 10 passages, and then grown in normal media. Expression of caveolin-1 and PTRF/cavin-1 was monitored by immunoblotting.

### Chemotactic Transmigration Assay

Corning transwell® inserts with polycarbonate filters with 8-µm pores were used to measure chemotactic migration. Cell suspensions prepared in serum free media were loaded on the top chamber, and serum-containing media in the lower chamber. NIH 3T3 cells were allowed to migrate for 2 hours towards 10% serum, while PC3 cells were allowed to migrate for 24 hours towards 20% serum in a humidified 37°C incubator. Filters were washed with cold PBS and then fixed with 4% paraformaldehyde. Filters were removed from the transwell®, the cell nuclei stained with DAPI and mounted onto slides. Nuclei were visualized by fluorescence microscopy and quantitated using Image J. The migration of DU145 cells was determined using a modified Boyden chamber assay as previously described [12] after determination of the serum concentration eliciting the best chemotactic response (0.5%).

### Immunofluorescence of Transmigrating Cells

A modified Boyden chamber was used to visualize caveolin-1 and PTRF in transmigrating cells. A suspension of 7,500 cells was loaded in the upper wells of a migration chamber prepared with 8-µm pores, polycarbonate filters coated with rat tail collagen type I. Migration was allowed to proceed for 3 h, and filters were processed for immune-staining as previously described [13]. Following incubation with the indicated primary antibodies, filters were incubated with biotinylated goat anti rabbit antibody and Texas-Red-Avidin from Vector (Burlingame, CA). Transmigrating cells were visualized using a Leica TCS SP5 confocal microscope.

### Immunofluorescence Microscopy

Cells were grown to 70% confluency on glass coverslips, washed in PBS and fixed in 2% PFA prior to permeabilisation with 0.1% Triton X-100. Coverslips were washed with 2% bovine serum albumin (BSA) in PBS then incubated with monoclonal anti-Rac1 antibody for one hour prior to washing and incubating with anti-mouse Alexa 594 antibody. Cells were washed and mounted for imaging. Fluorescence was imaged using a Zeiss LSM510 Meta Duoscan with Zen imaging software and processed using Adobe Photoshop CS2 for images and Image J for quantitation. Images for quantitation were captured at a 1.38AU pinhole size to ensure entire cell fluorescence was captured. Twenty cells for each condition were evaluated for fluorescence intensity distribution. A straight line was drawn across the length of each cell and average fluorescence intensity measured along the cell length using Image J.

### Video Microscopy

Cells for real-time microscopy were plated onto 35 mm glass-bottom tissue culture dishes (MatTek Corp.) 48 hrs prior to imaging. A scratch wound was made in the cell monolayer immediately before imaging and the cells were transferred into CO_2_-independent medium supplemented with 0.1% BSA (Roche Diagnostics, IN, USA). Cells were imaged at 37°C using an Olympus IX81 inverted microscope fitted with an OBS Xenon lamp and 10× objective, Solent Scientific incubation chamber and Olympus F-View II monochrome CCD camera. Time series images were collected at 1 frame every 5 min for up to 12 hr using the RFP/GFP excitation filter with a laser intensity of 2%. All images were converted to 16-bit TIFF files and further analysed using Image J software (National Institutes of Health, Bethesda, MD). QuickTime movies were assembled using Image J 1.37p and still images were compiled using Adobe Photoshop CS3.

Analysis of cell projections and cell shape was performed independently by 3 researchers blinded to experimental groups and the results were pooled.

## Supporting Information

Video S1 **3D reconstruction of migrating PTRF-GFP PC3 cell stained for caveolin-1 (red).**
(AVI)
